# Address of Dr. W. W. Allport, before the Illinois State Microscopical Society

**Published:** 1869-08

**Authors:** 


### ADDRESS OF DR. W. W. ALLPORT, BEFORE THE ILLINOIS STATE MICROSCOPICAL SOCIETY.

Ladies and Gentlemen:—The character, as well as the large number present this evening, indicates an interest felt by the most respectable and influential portion of our citizens in "The State Microscopical Society of Illinois," hardly hoped for by its projectors and those who were the most instrumental in its organization. In view of the manifest interest felt in this society, I have been requested to state to you its origin, present condition and objects, as well as some of the uses of the microscope. In the early part of the past winter a circular was issued from "The Chicago Academy of Natural Sciences," inviting all those in the city who took an interest in microscopical investigations to meet at their rooms for the purpose of organizing a microscopical section to the academy. Agreeably to this invitation, quite a respectable number of gentlemen met. At this meeting a diversity of opinion existed. Some were in favor of a society that should work in connection with the academy, while others wished a separate organization. A committee was appointed to take the matter under consideration, and to report their conclusions, and a plan for organization at a subsequent meeting. After holding several meetings, at which the report of the committee, and the views of the various gentlemen present, were fully discussed, it was thought that an independent society could be more easily managed, and that more good could be accomplished by it than by working as an adjunct to any other society. As a result of this conclusion a temporary organization, which has been known as "The Chicago Microscopical Club," was formed. A bill was immediately prepared and sent to our State Legislature, and a law was passed incorporating "The State Microscopical Society of Illinois," under which act of incorporation we are now organized and acting, and into which the Chicago Microscopical Club has been merged. From the statement I have made you will see that our society is but a few months old, and yet I am pleased to state that we have some sixty resident members, among whom are some of the leading members of the medical profession and the best amateur microscopists of the city and State. Hardly a meeting has passed recently at which we have not received donations to our cabinet, either from our own members or from microscopists residing in different parts of the country, some of which, as you will see, are exceedingly rare, beautifully prepared, and artistically mounted. We are also receiving offers of exchanges and donations from prominent microscopists and the officers of kindred associations not only at home, but abroad. The proceedings of some of our meetings have been published in the scientific journals of Europe, as well as of our own country. From the past, the society has every reason to be encouraged and to look with hope to the future ; expecting, as it does, that its list of resident working members will be largely increased at our next meeting, which, in accordance with the provisions of our by-laws, will not occur until the first Friday in October. In addition to resident membership the by-laws of the society provide for honorary, associate and corresponding memberships. And, while we intend to be somewhat liberal in the admission of resident members, care will be exercised to admit none to the other memberships whose name would not be an honor to the society, or whose contributions will not increase the interest of its meetings, and the usefulness of the society. The leading object of the society will be the cultivation of microscopy in the investigation and demonstration of scientific subjects. Committees have been appointed for the special systematic investigation of floral structures, infusoria, cryptogamous plants, vegetable and animal histology, vegetable and animal pathology, vegetable and animal parasites, crystallography, and kindred branches, during the ensuing year. Besides which it is desired to make the microscope useful in social and commercial interests, by detecting adulteration in food and fraud in fabrics, and to exhibit, from time to time, so far as may be possible, to such of our citizens as may appreciate it, the minute handiwork of our Creator, as it can be seen in no other way than through the almost infinite vision of the microscope. In all His works, however we may enlarge or refine our vision, we find nothing common or unworthy our notice. The eager traveler crosses oceans and continents, climbs rocks and mountains, that he may gaze upon the beauties and wonders of the landscape; unmindful, too often, of the not less wounderful creations that surround his path and are crushed beneath his feet. The way-side flower, the springing leaf, the tuft of moss, the blade of grass, or tiniest insect reveals, under the microscope, colors that contest in brilliancy and beauty with the rainbow and sunset, and forms of more subtle grace and delicate tracery than sculptors have ever chiselled or artist's pencil can hope to rival. There are few things about which the public have a more erroneous impression than the use of the microscope. The popular notion that microscopy is one of those abstruse sciences, the acquisition of which requires years of study and patient practice, is an error, as it is a simple art, easily acquired. The microscope is merely an instrument of observation. It sharpens the eye, and peers into everything. No invention of man has a wider range of application in its uses. It should be the companion not only of the scientific, but it might, with profit, be found in every counting-room, work shop and household. As an instrument of education it has no equal, and no school room should be without it. From the fact that the microscope has been so extensively used by scientific men, let no one suppose that it is for the exclusive use of gentlemen. The ladies, with their quick perceptions, refined organism, and delica y of touch, are peculiarly adapted to manipulate both instrument and objects, and there is no reason why practice would not render them skillful microscopists. Besides the instruction and personal pleasure there would be derived from the use of this instrument, ladies would find it to be an easy and agreeable way of entertaining their friends. They would also find many valuable hints in the delicate tracery of minute organisms, visible only under the microscope, which would suggest beautiful designs for their embroidery and fancy work, and its skillful management by them might with propriety be regarded as a refined accomplishment. As a teacher of theology this brazen tube has no rival. No doctor of divinity, however learned and eloquent, can so successfully urge the fallacy and utter absurdity of atheism, and route with shame and confusion the advocates of infidelity. For it reveals in every atom of the universe that "that the hand that made us is divine." So "he that hath eyes to see let him see," and in the beautiful lines of Young so appropriately placed on the programme of the evening by our efficient Secretary:

"Think naught a trifle, though it small appear;
Small sands the mountain, moments make the year;
And trifles life."

